# A novel, microfluidic high-throughput single-cell encapsulation of human bone marrow mesenchymal stromal cells

**DOI:** 10.1007/s10856-024-06785-z

**Published:** 2024-03-25

**Authors:** Narjes Rashidi, Alex Slater, Giordana Peregrino, Matteo Santin

**Affiliations:** 1 Centre for Regenerative Medicine and Devices, University of Brighton, Huxley Building Lewes Road, Brighton, BN2 4GJ UK; 2 School of Applied Sciences, University of Brighton, Huxley Building Lewes Road, Brighton, BN2 4GJ UK

## Abstract

The efficacy of stem-cell therapy depends on the ability of the transplanted cells to escape early immunological reactions and to be retained at the site of transplantation. The use of tissue engineering scaffolds or injectable biomaterials as carriers has been proposed, but they still present limitations linked to a reliable manufacturing process, surgical practice and clinical outcomes. Alginate microbeads are potential candidates for the encapsulation of mesenchymal stromal cells with the aim of providing a delivery carrier suitable for minimally-invasive and scaffold-free transplantation, tissue-adhesive properties and protection from the immune response. However, the formation of stable microbeads relies on the cross-linking of alginate with divalent calcium ions at concentrations that are toxic for the cells, making control over the beads’ size and a single-cell encapsulation unreliable. The present work demonstrates the efficiency of an innovative, high throughput, and reproducible microfluidic system to produce single-cell, calcium-free alginate coatings of human mesenchymal stromal cells. Among the various conditions tested, visible light and confocal microscopy following staining of the cell nuclei by DAPI showed that the microfluidic system yielded an optimal single-cell encapsulation of 2000 cells/min in 2% w/v alginate microcapsules of reproducible morphology and an average size of 28.2 ± 3.7 µm. The adhesive properties of the alginate microcapsules, the viability of the encapsulated cells and their ability to escape the alginate microcapsule were demonstrated by the relatively rapid adherence of the beads onto tissue culture plastic and the cells’ ability to gradually disrupt the microcapsule shell after 24 h and proliferate. To mimic the early inflammatory response upon transplantation, the encapsulated cells were exposed to proliferating macrophages at different cell seeding densities for up to 2 days and the protection effect of the microcapsule on the cells assessed by time-lapse microscopy showing a shielding effect for up to 48 h. This work underscores the potential of microfluidic systems to precisely encapsulate cells by good manufacturing practice standards while favouring cell retention on substrates, viability and proliferation upon transplantation.

**Graphical Abstract:**

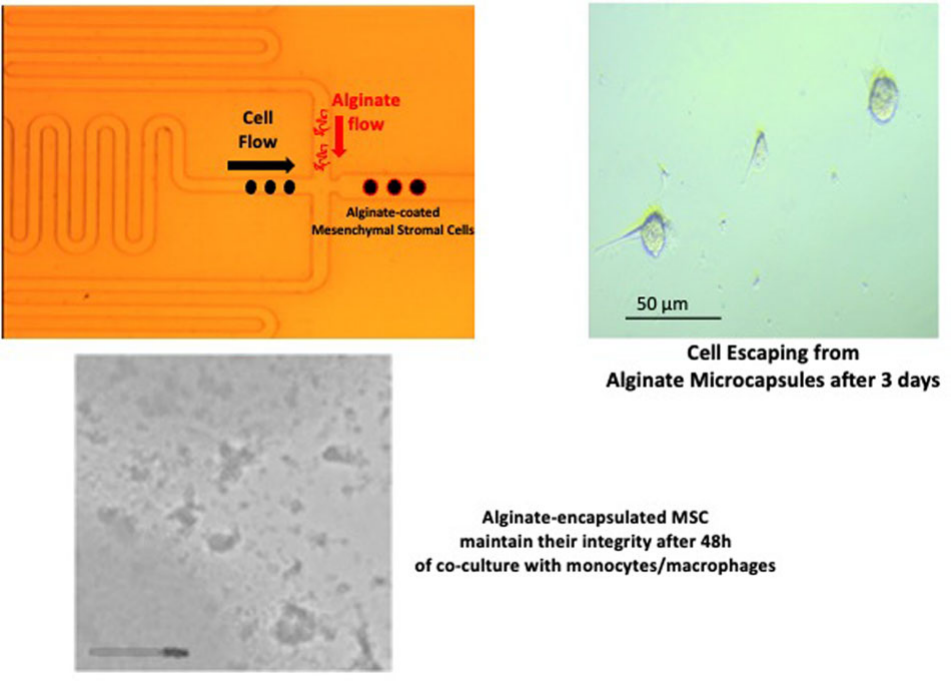

## Introduction

Human bone marrow mesenchymal stromal cells (MSCs) have been used in clinics for cartilage repair in osteoarthritic conditions and have more recently been proposed as a promising therapeutic intervention for myocardial infarction [1–3]. In the case of the treatment of knee osteoarthritis, a total of 23 non-case report studies have been reported. Thirteen of them were designed as randomized controlled studies, although with heterogeneity in source cells, preparation methods, and dosage of MSCs [1]. The single injection dosage ranged from 1.56 × 10^4^ to 1 × 10^8^ cells, and the most wildly proved dosage was 5 × 10^7^ cells [1]. Repeated injections or high dosage showed superiority over single injection or low dosage [4], with mild adverse events being transient arthralgia, swelling of joints after local injection and low back pain which spontaneously relieved within 7 days suggesting an early inflammatory response to the transplanted cells [5, 6].

Myocardial infarction often results in irreversible loss of cardiomyocytes, leading to impaired cardiac function and potentially life-threatening consequences. It has been shown that MScs contribute to myocardial repair through paracrine mechanisms, immunomodulation and anti-inflammatory effects [7]. These unique properties make MSCs an attractive candidate for cell-based therapies aimed at restoring cardiac structure and function post-myocardial infarction.

However, the use of MSCs in clinics is limited by the availability of carriers that are compatible with a minimally-invasive procedure (i.e., injection) and that can enable the cells to be retained at the site of treatment while being protected from the inflammatory environment dominating osteoarthritis and acute myocardial infarcted areas.

In this respect, cell encapsulation within microgels, as opposed to biomaterial scaffolds which are not suitable for injection procedures, has emerged as a transformative approach in various fields, including pharmaceutical research, tissue engineering and regenerative medicine [8–12]. These microgels are suitable for precise manufacturing and serve as discrete 3D microenvironments to preserve the phenotype of the cells and their controlled delivery to the site of transplantation. In particular, the encapsulation of living cells in micrometer-sized alginate particles has gained significant attention due to its potential applications in studying cell behavior, tissue repair, and the development of tissue-mimicking structures [13, 14]. However, to fulfill the therapeutic potential of cell encapsulation within microgels, several challenges must be addressed. These challenges include achieving homogenous network structures within the microgels, controlling particle size and size distribution, and ensuring efficient oxygen and nutrient exchange for encapsulated cells [15–17].

Alginate microgel particles are typically produced by emulsification in an aqueous solution of alginate in an oil phase and the consequent crosslinking through ionic bonding with divalent ions like Ca^+2^. This ionic crosslinking process initiates promptly upon contact between alginate chains and calcium ions. This rapid reaction often leads to uncontrolled gelation which, when applied to advanced manufacturing methods such as microfluidic devices, can result in issues such as channel clogging and nonuniform droplet formation [15, 18]. Previous research tried to address these challenges by separating the droplet formation process and the calcium-driven crosslinking to prevent unintended gelation before droplet formation [19]. This technique is also hindered by two key challenges: (i) the necessary drop in pH for alginate gelation and (ii) the subsequent removal of the oil phase and unreacted calcium ions. These hurdles significantly prolong the process, jeopardizing cell viability and operational efficiencies. To address these issues, the present work aimed at developing a microfluidic system for the single-cell encapsulation of MSCs where aqueous alginate solutions of varying concentrations were integrated with flowing MSC suspensions; the system relying on the ability of the alginate hydroxyl groups to establish immediate hydrogen bonds with the cell glycocalyx upon contact of the two fluids thus eliminating the need for the use of cell-incompatible oil phase and calcium crosslinking.

The properties of the encapsulated cells to adhere on solid surfaces and to be protected from the attack of proliferating monocytes/macrophages within 48 h were studied alongside their ability to escape the capsule and proliferate over time.

## Materials and methods

### MSCs’ culture

Human bone marrow MSCs (2 donors, male and female, 18- and 25-year-old Lonza, UK) were cultured in a chemically-defined medium (Lonza’s TheraPEAK^TM^ MSCGM^TM^ Mesenchymal Stem Cell Growth Medium). To preserve MSCs stemness, cells were subjected to no more than 2 passages prior to their encapsulation.

### MSCs’ microfluidic encapsulation

**Fig. 1:**
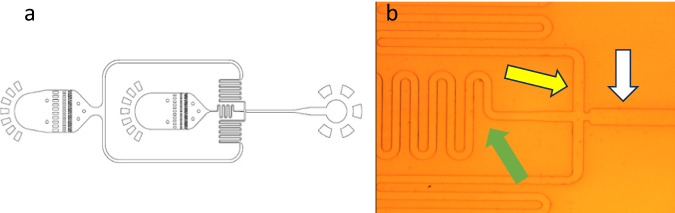
Microfluidic chip geometry. **a** schematic illustration of the flow-focusing microfluidic chip. **b** microscopic image of the microchip for single-cell encapsulation, green arrow: central channel for cell flow. yellow arrow: alginate-dispensing channel. white arrow: outlet channel

An Elveflow (France) microfluidic system was used with precise pressure control to inject fluids in microchannels. The system consisted of a microfluidic controller that generates controlled air pressure applied to the system fluid reservoirs and inlets. All the components of the microfluidic system (i.e., tubing and the microfluidic chip) were disinfected by a 70% v/v ethanol/water flow for 1 h. Air was also filter-sterilized applying 0.2 μm filters sterile at both the alginate and cell suspension tubing inlets. Cells were suspended in the same chemically-defined, serum-free medium used for their culturing at different densities (from 1 x 10^5^ to 1 x 10^6^ cells/ml). Alginate (Alginic acid sodium salt, Mw: 120K–190K Daltons, Sigma Aldrich, UK) water solutions at 1%, 2% and 3% w/v were prepared in sterile conditions. The tube containing the cells was connected to the Darwin Microfluidics DG-DM-60/1.2 mm Droplet Generator (Fig. 1a) and cells in the tube were kept in suspension by gentle stirring with an orbital shaker. Upon application of a constant air pressure of 300mbar, the MSCs were injected from the tube into the microfluidic reservoir that avoids cells aggregation and enables the introduction of single separated cells into the central channel (Fig. 1b, green arrow) where they were exposed to the flow of the alginate aqueous solution injected through the perpendicular channel at a pressure of 400 mbar (Fig. 1b, yellow arrow). The microbead-encapsulated MSCs were collected from the microfluidic chip outlet into a sterile Eppendorf tube and kept in suspension until used for characterization or biological experiments within 30 min.

### Encapsulated MSCs characterization

Single-cell MSCs’ encapsulation was assessed by DAPI staining of the nuclei of the cells immediately after their transfer to a 24-well plate (200 μl of cell suspension/well). The percentages of free and encapsulated cells were quantified by cell counting from 3 different wells and expressed as a percentage of encapsulation.

The diameters of the MSCs encapsulated at different concentrations of alginate were measured and compared to those of control MSCs. The average size of microbeads was determined by measuring the diameter of 30 microparticles by the Image J software. To obtain accurate size measurement, the images were properly calibrated by converting pixels to microns based on the known image scale bar. Data were expressed as mean diameter (μm) standard deviation from *n* = 30 different samples.

### Microcapsule adhesion to tissue culture plastic and MSC viability

The adhesive properties of the encapsulated cells as well as the MSC’s ability to escape the capsule and maintain their own adhesive properties, viability and proliferation were tested by their seeding at a density of 2 x 10^5^ cells/ml on adherent tissue culture plastic (Falcon, USA) and incubation in the same chemically-defined medium at 37 ^o^C, 95% air 5% CO_2_, static conditions, over 3 days. The process of cell liberation from microcapsules was monitored by a Lux time-lapse light microscopy (Axion Biosystems, UK) and later by higher magnification light microscopy.

### In vitro model of encapsulated MSCs protection from proliferating inflammatory cells activity

The ability of alginate-encapsulated single MSCs (1 × 10^5^ cells/ml) to preserve their integrity upon an insult by inflammatory cells was assessed by their co-culturing with U937 monocytes/macrophages cell lines (ECACC general cell collection, Cat number 85011440) seeded at different concentration (2 × 10^5^ to 5 × 10^5^ U937/mL) over 2 days. The morphological integrity of the encapsulated cells upon monocytes/macrophages proliferation and activation was qualitatively analysed by time-lapse microscopy.

## Results

### Optimization of the experimental process

The microfluidic system was optimized considering a range of parameters including chip geometry, applied air pressure, cell number and the concentration of the alginate solution.

**Fig. 2:**
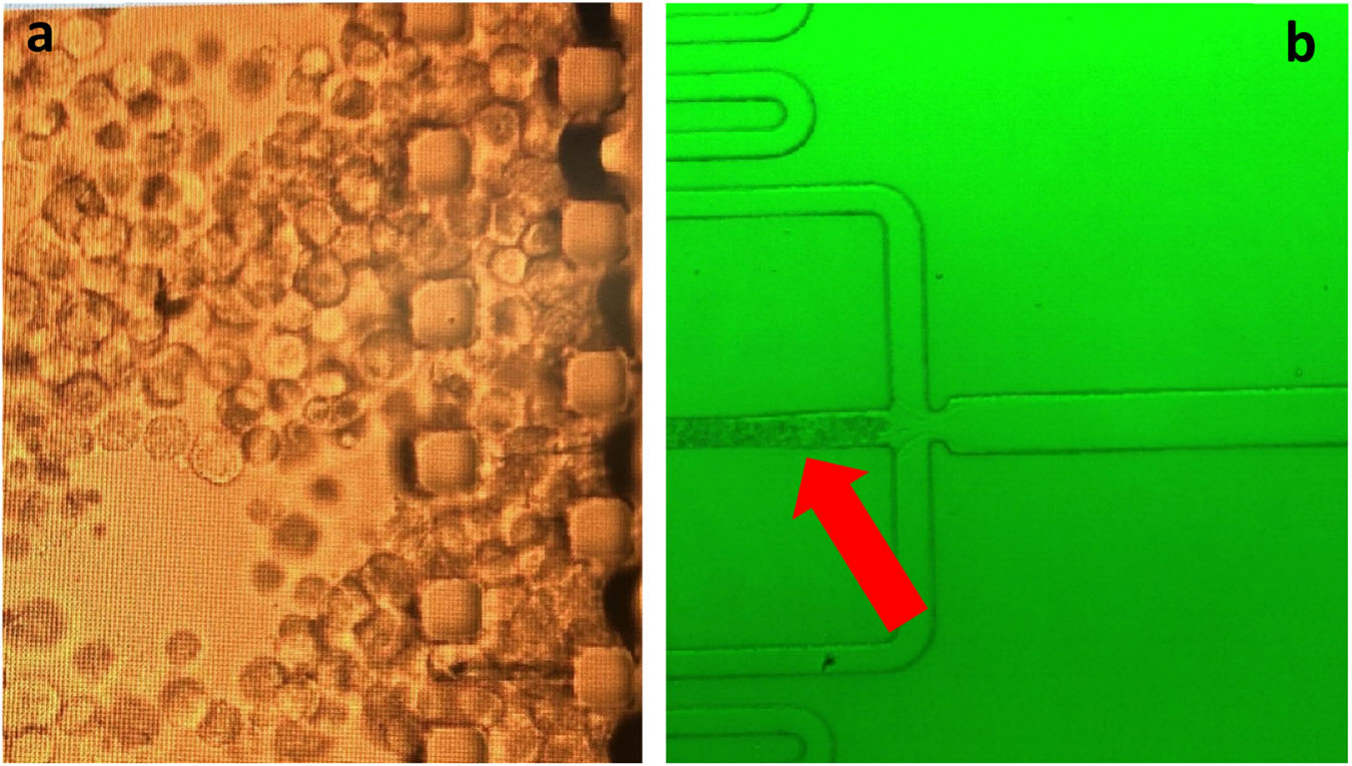
Microfluidic compartment blockage upon injection of cell suspension of density (1 × 10^6^ cell/ml). Cell aggregation in **a** the chip reservoir and **b** the central channel. Red arrow: typical case of cell blockage in the central channel upon high cell density flow. Images taken by velocity camera

The serpentine design of the chip central channel used in this study showed to be particularly advantageous for cell suspension flow as the extended channel length allows for a more gradual change in the flow direction, reducing pressure drop and minimizing the risk of channel blockages by cell aggregation. Likewise, the injection of high cell numbers into the system tend to accumulate cells in the microfluidic reservoir causing its clogging as well as the irregular injection of cells through the central channel thus impacting on the reproducibility of the encapsulation process (Fig. 2a, b). This was not the case when the cell number was reduced (data not shown). To this end, the minimum cell density found to yield a reliable process was 1 × 10^5^ cells/ml. Likewise, among a range of air pressure conditions tested, the ideal channel pressure was determined to be 400 mbar in the alginate channel and 300 mbar in the cell suspension channel.

**Fig. 3:**
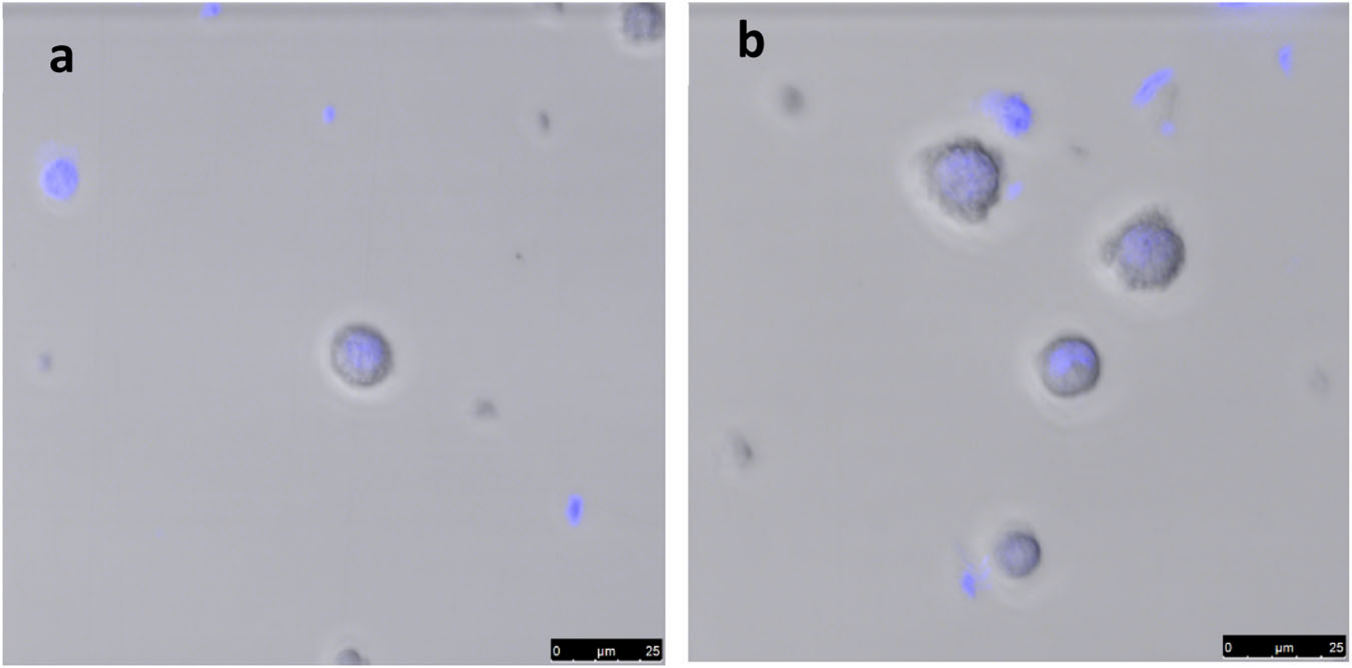
MSC encapsulation in coatings of varying alginate concentrations. **a** MSC encapsulation in alginate 1% w/v and **b** 2% w/v. Images show merged bright field and epifluorescence confocal microscopy of encapsulated MSC stained by DAPI

The optimal alginate concentration was identified as 2% w/v. While 1% w/v alginate solution showed a reduced percentage of encapsulated cells (46%, Fig. 3a), MSC encapsulation in 2% w/v alginate yielded a percentage of encapsulated cells of 63% (Fig. 3b) in 30 min with the most reproducible microcapsule morphology. Experiments performed using 3% w/v alginate resulted in non-reproducible alginate beads probably caused by the more viscous properties of the biopolymer solution (data not shown).

### Size measurements and alginate film thickness

**Fig. 4:**
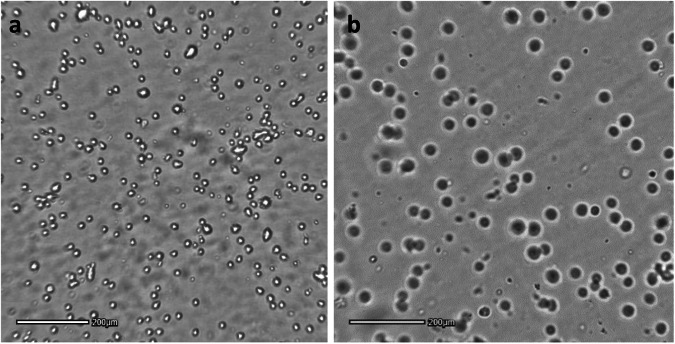
Light microscopy of **a** uncoated cells, **b** 2% w/v alginate-encapsulated MSCs. The plane of focus of the microscope shows that uncoated cells were still floating within 2 h after seeding in tissue culture plate, while coated ells showed larger diameter and all rapidly adhering and remaining stable at the bottom of wells. Images taken by Axion wireless microscope

The average size of MSCs encapsulated in 2% w/v alginate was found to be 28.2 µm ± 3.7 whereas the average size of uncoated MSCs was 9.3 µm ± 1.8 (Fig. 4a, b). Thus, in the case of the MSCs encapsulated with 2% w/v alginate the indirect estimate of the coating average thickness was 18.9 µm, while in the case of cells encapsulated with 1% w/v alginate the estimated coating thickness was 5 µm raising concerns about the potentially limited adhesive and immune-protective potential of the coating.

### Encapsulated MSCs adhesive and proliferative properties

**Fig. 5:**
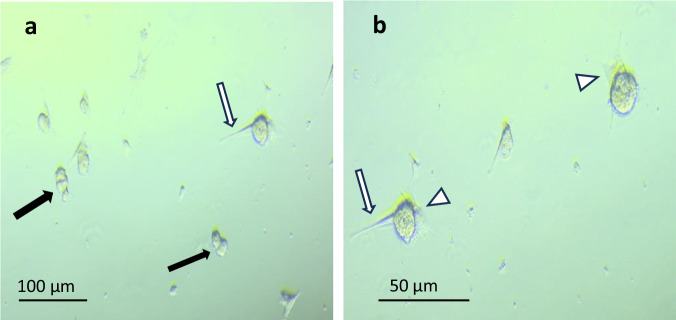
Light microscopy of 2% w/v alginate-encapsulated MSC after 3 days of culturing on tissue culture plates in chemically-defined medium. **a** 10 x magnification **b** 20 x magnification. MSCs showed to evade their alginate coating establishing interactions with the plastic substrate through filopodia (white arrows) and lamellipodia (arrowheads) and showing sign of mitotic division (black arrows)

When seeded on adherent polystyrene tissue culture plastic, MSCs encapsulated in 2% w/v alginate resulted deposited at the bottom of the well within a few minutes when compared to uncoated cells that remained floating for hours and showed to be static over the incubation time (Fig. 4a, b). The adhesive property of the alginate capsule was confirmed by light microscopy analysis at higher magnification over 3 days of incubation where encapsulated cells were shown to initiate a process of evasion from their alginate microcapsules with the protrusion of filopodia (Fig. 5a, b, arrows) and more established lamellipodia (Fig. 5a, b, arrowhead, Supplementary Material Video 1). Noticeably, the encapsulated MSCs showed to have maintained their proliferative potential as mitotic processes leading to cell division appear to take place from day 3 (Fig. 5a, black arrows, Supplementary Material Video 1).

### Encapsulated MScs interaction with immune cells

**Fig. 6:**
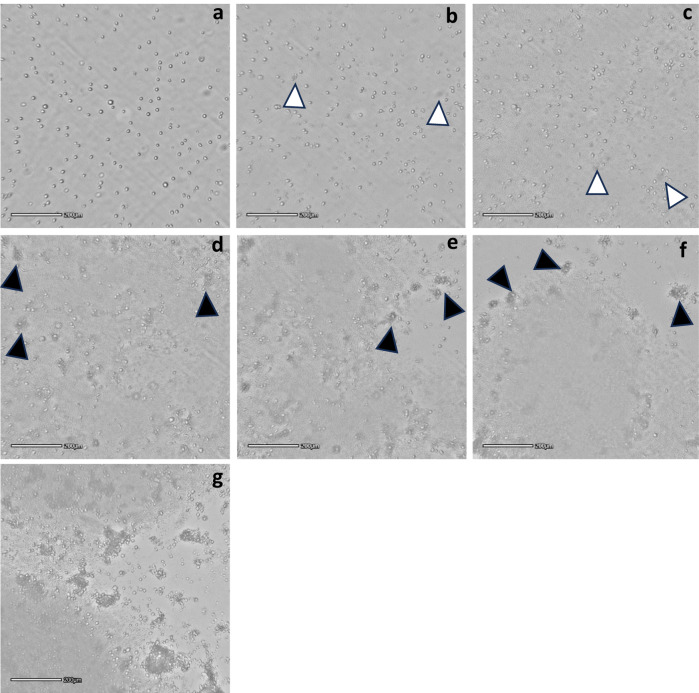
Immune cells interaction with encapsulated MSCs over 48 h of exposure. **a** time 0, **b** 8 h; macrophages cluster formation (white arrowheads), **c** 14 h; macrophage cluster aggregation in proximity of MSC-encapsulating microbeads becoming visible (white arrowheads), **d** 24 h; proliferating macrophages start forming denser clusters around encapsulated cell microbeads (black arrowheads), **e** 36 h; microbeads are increasingly surrounded by immune cell clusters (black arrowheads) while maintaining their stability and integrity, **f** and **g** 48 h; proliferating immune cells cover most of the tissue culture plastic surface (central grey area) and large clusters almost completely envelop the microbeads that still preserve their intact structure (black arrowheads). **g** zoomed image of an 2% w/v alginate-encapsulated MSC surrounded by clusters of U937 cells showing microcapsule still intact

Time-lapse microscopy analysis (Fig. 6a–f, Supplementary Material, Video 2) showed the protective effect of the 2% w/v alginate on MSCs when co-cultured with U937 monocyte/macrophage cell lines at different cell seeding densities. Irrespective of their seeding density, the immune cells initiated to attack the encapsulated MSCs approximately 7–8 h after exposure while showing signs of adhesion onto the tissue culture plastic and relatively rapid proliferation (Fig. 6d–f, black arrowheads; Supplementary Material, Video 2, dark large expanding areas observed at the video later stage). These immune cells encircled the encapsulated MSCs that remained structurally stable and intact even after 48 h of exposure and regardless of the initial U937 seeding density. While aiming at mimicking the inflammatory conditions of osteoarthritis or acute myocardial infarction it is important to note that, in terms of inflammatory cell numbers, the in vivo conditions are likely to be milder than those adopted in this in vitro model. Indeed, the normal synovial fluid contains less than 200 leukocytes/ml, whereas in acute phases of inflammatory arthropathies, the leukocyte count significantly increases to 50,000/ml or more [20], which is ten times lower that the cell density used in this study.

## Discussion

Cell therapy has been advocated as the future alternative to the treatment of tissues damaged by trauma or diseases [21]. Among the various cell phenotype studied as therapeutics, MSC are widely advocated for their multipotency making them able to differentiate in different types of tissue cells as well as to contribute to tissue regeneration by releasing key paracrine factors (e.g. the vascular endothelial growth factor, VEGF) to the tissue cells [22, 23]. However, the translation of this widely accepted therapeutic potential from concepts and pre-clinical studies to clinical applications is restrained by a range of limitations that so far have prevented their approval by the regulatory bodies [23]. From a regenerative point of view, human bone marrow MSC are known to be able to differentiate into three main lineages, adipocytes, chondrocytes and osteoblasts, but their ability to differentiate into nerve cells, glioma cells and skeletal muscle cells has been demonstrated in vitro upon specific culturing conditions [23]. However, those favorable differentiation conditions are not available in the complex microenvironment of a damaged tissue limiting, alongside their well ascertained slow rate of proliferation, their therapeutic potential [21, 23]. To this end, MSC-based therapy has been mainly pursued through strategies that are based either on their genetic manipulation or combination with biomaterial carriers [21, 24]. Among them, alginate, a polysaccharide made of glucuronic and mannuronic acid and found in the cell walls and intracellular spaces of brown seaweed, has been widely studied because of its overall biocompatibility that includes non-toxicity, non-immunity, extracellular matrix biomimetic features and biodegradability [25]. This relatively inexpensive biomaterial also offers relatively low costs of sourcing and suitability for engineering into hydrogels, microspheres, microcapsules, sponges, foams and fibers [26]. These properties make the use of this biomaterial as a carrier for MSC attractive, particularly in those clinical applications where minimally-invasive procedures, the control of cell phenotype and protection in highly inflamed tissue are needed. These include, among others, the treatment of mild/moderate osteoarthritis conditions and myocardial infarction acute phase where a transplantation by localized injection is desirable [27, 28]. MSC therapy for the treatment of osteoarthritic joints based on intra-articular administration as well as for acute myocardial infarction have been studied in several clinical trials showing different outcomes that suggest that the procedure is still far from its optimization and that repeated doses of cells may be required over time [4, 27, 28]. Such an approach rules out the administration of the cells in combination with biomaterial scaffolds unless injectable self-setting formulations are used. While having the potential of providing the cells with a microenvironment adequate for their retention at the site of implantation, viability and phenotype preservation (or differentiation) and protection from inflammatory insults, it is argued that the reproducibility of these procedures may be affected by phase separation upon injections, gelation kinetics, uneven distribution of cells within the construct and lack of control over oxygen, nutrients and catabolites diffusion [29]. Encapsulation of cells in beads such as alginate beads is also prone to drawbacks hindering their use in clinics [30]. These drawbacks include immunogenicity caused by lipopolysaccharide contaminants not efficiently removed during the alginate purification, crosslinking agents that can be toxic to the cell or immunogenic, and the thickness of the capsule that may limit the diffusion or the protection of the cells in the bead core [30]. To this end, the present work has focussed on the ability of producing a highly reproducible, high throughput method of alginate thin-coating deposition at the surface of MSC by the accurate control of a microfluidic device. The data obtained confirmed the research hypothesis that the highly hydrophilic nature of both the alginate and the cell glycocalyx could led to the formation of a thin and stable coating that could limit the size of the beads to a diameter feasible for injection through endoscopy while increasing the cell rapid attachment to surfaces. The use of a microfluidic system with a T-junction and flow-focusing geometry to achieve MSC encapsulation has previously been pursued demonstrating that T-junction coaxial capillary, micro-nozzle cross-flow system, the size of the orifice of the T-junction and biomaterial viscosity all affect the size of the droplets formed [31, 32]. These studies have been focussing on the encapsulation of multiple, not single, MSC in protein-based microcapsule including poly-L-Lysine and methacrylated gelatine that do not have the desired characteristics in terms of specific clinical applications and immunogenic potential [33–35]. The choice of a serpentine geometry of the microfluidic chip, the testing of different cell suspension densities and alginate concentrations considered in this study offer a significant step forward to the development of MSC encapsulation protocols that fulfills most of the parameters required for both manufacturing and clinical requirements. Furthermore, the demonstration of the cells to maintain their viability, adhesive properties and proliferation, alongside their protection upon an inflammatory insult, provides further evidence about the potential of this method to be applied to future in vivo and clinical studies.

## Conclusion

The present study demonstrates the significant potential of employing microfluidic technology for precise MSCs’ single-cell encapsulation process in alginate thin coating that fulfills the standard requirement for advanced therapy medicinal products (ATMP). Together with the FDA approval of alginate for medical applications available since the 70 s and the data emerging from clinical studies where this hydrogel has been tested for cardiac applications [36], the present study shows that the speed, scaled and reproducibility of the preparation obtained by a compact microfluidic system, easy to accommodate in a confined space of a clean room or even in a surgery theater, may pave the way towards an alternative, cost- and clinical effective way to apply MSC therapy in the treatment of clinical conditions where minimally-invasive and rapid procedures are advocated.

## Supplementary Information

Rashidi_et_al_JMSMM_2023_Video1

Rashidi_et_al_JMSMM_2023_Video2
